# Comment to Ruiyu Liu et al.: Comparative analysis of gene expression profiles in normal hip human cartilage and cartilage from patients with necrosis of the femoral head

**DOI:** 10.1186/s13075-016-1110-2

**Published:** 2016-10-13

**Authors:** Zeling Long, Tianlong Huang, Wanchun Wang

**Affiliations:** The Second Xiangya Hospital of Central South University, Changsha, China

It was with great interest that we read the study entitled “Comparative analysis of gene expression profiles in normal hip human cartilage and cartilage from patients with necrosis of the femoral head (NFH)” by Ruiyu Liu and colleagues published in *Arthritis Research & Therapy* in May 2016 [1].

The authors identified a set of differentially expressed genes which may contribute to the destruction of articular cartilage in patients with necrosis of the femoral head (NFH). Specimens were collected from 18 patients with non-traumatic NFH, classified by the Ficat system as grade III, and 18 healthy control subjects. However, the paper did not provide sufficient information about the main causes of NFH. Thus, the conclusion that the cartilage damage in NFH is related to the differential expression of these genes is debatable. The causes of non-traumatic NFH are complicated. A nationally representative survey of 30,030 people demonstrated that blood levels of triglycerides, total cholesterol, LDL-cholesterol, non-HDL-cholesterol, male gender, urban residence, family history of osteonecrosis of the femoral head, heavy smoking, alcohol abuse and glucocorticoid intake, overweight, and obesity were all significantly associated with an increased risk of non-traumatic NFH [2]. Some of these factors may also result in differential gene expression in articular cartilage. A study by Sniekers et al. [3] showed that estradiol affects the expression of anabolic and catabolic genes (e.g., those encoding aggrecan (AGC)1, matrix metalloproteinase (MMP)2, MMP14, and tissue inhibitor of metalloproteinase (TIMP)2, transforming growth factor (TGF)B2, and TGFB3) in bovine cartilage explants. Another study showed that leptin, a hormone associated with obesity, induces ADAMTS-4, ADAMTS-5, and ADAMTS-9 gene expression through mitogen-activated protein kinase and NF-ĸB signaling pathways in human chondrocytes [4]. Considering these issues might help to avoid irrelevant variables. The control group should be in accord with the NFH group and the established causes of NFH, such as estrogen in-take history, alcohol abuse, obesity, and so on. In addition, it would be better to provide basic information like height, habits, or history of drug use.

Another concern is that the methods for both selecting genes for quantitative RT-PCR validation and the immunohistochemical analysis might not been explained clearly. The nine genes were not the most significantly differentially expressed according to the gene expression ratios in Table 2 showing the significance analysis of microarrays (SAM) results of the patients with NFH. We would suggest selecting the genes with extreme gene expression ratio values from the nine functional groups mentioned in Table 2. Moreover, the microarray analysis may be more convincing if the sample size could be increased.

In this study, it remains unclear whether the changes in gene expression lead to the cartilage damage in NFH. To clarify their role, we suggest an additional animal experiment to study the dynamic change in gene expression and loss of cartilage in models at different stages of NFH, which may help to clarify the mechanisms by which the identified genes affect the development of NFH.

## Abbreviations

NFH: necrosis of the femoral head
qRT-PCR: quantitative real-time PCR
