# Supplementary material for: Comment to Ruiyu Liu et al.: Comparative analysis of gene expression profiles in normal hip human cartilage and cartilage from patients with necrosis of the femoral head
Source: Arthritis Res Ther. 2016 Oct 13;18:238. doi: 10.1186/s13075-016-1110-2 (PMC5062942; doi:10.1186/s13075-016-1110-2)
Supplement: Related texts

# Reply: Authors’ response

**Authors:** Ruiyu Liu, Qi Liu, Kunzheng Wang, Xiaoqian Dang, Feng Zhang

We thank Long and colleagues for their interest in our recent publication [1]. We would like to respond to their comments.

First, we totally agree with the comment that non-traumatic necrosis of the femoral head (NFH) is a complex disease, mainly caused by excessive steroid use, alcohol drinking, as well as idiopathic NFH without certain etiological factors [5]. Additionally, heavy smoking, overweight, and hyperlipemia were reported to contribute to the development of NFH. To control the potential impacts of steroids, alcohol, and other confounding factors on our study results, all case samples were idiopathic NFH patients without histories of steroid and alcohol abuse, heavy smoking, hyperlipemia, and obesity. The healthy control samples were matched to case samples for age and sex and also did not have histories of steroid and alcohol abuse, heavy smoking, hyperlipemia, and obesity.

Second, Long and colleagues suggest selecting the genes with extreme expression ratios for quantitative real-time PCR (qRT-PCR) validation. We think that it was better to select genes by considering both expression ratios and biological relevance to NFH. The selected nine qRT-PCR validation genes were not only significantly differentially expressed between NFH and controls but also generally had evidence supporting their roles in the normal growth, homeostatic maintenance, or damage of cartilage. Additionally, we agree with their comment that using more cartilage samples could increase the accuracy of the microarray studies. However, it is usually difficult to collect suitable cartilage samples in practice, especially healthy cartilage samples. The same sampling design has been widely used in previous microarray studies of cartilage [6].

Third, Long and colleagues advise us to conduct animal experiments to clarify the differences between gene expression patterns at different stages of NFH. This is a good suggestion and will be performed in our future studies.
